# Margins of postural stability in Parkinson’s disease: an application of control theory

**DOI:** 10.3389/fbioe.2023.1226876

**Published:** 2023-09-14

**Authors:** Zahra Rahmati, Saeed Behzadipour, Ghorban Taghizadeh

**Affiliations:** ^1^ Mechanical Engineering Department, Sharif University of Technology, Tehran, Iran; ^2^ Djawad Movafaghian Research Center in Neurorehab Technologies, Sharif University of Technology, Tehran, Iran; ^3^ Rehabilitation Research Center, Department of Occupational Therapy, School of Rehabilitation Sciences, Iran University of Medical Sciences, Tehran, Iran

**Keywords:** margin of stability, Nyquist stability criterion, quiet stance posturography, postural control model, Parkinson’s disease, gain and phase margin, balance training

## Abstract

**Introduction:** Postural instability is a restrictive feature in Parkinson’s disease (PD), usually assessed by clinical or laboratory tests. However, the exact quantification of postural stability, using stability theorems that take into account human dynamics, is still lacking. We investigated the feasibility of control theory and the Nyquist stability criterion—gain margin (*GM*) and phase margin (*PM*)—in discriminating postural instability in PD, as well as the effects of a balance-training program.

**Methods:** Center-of-pressure (COP) data of 40 PD patients before and after a 4-week balance-training program, and 20 healthy control subjects (HCs) (Study1) as well as COP data of 20 other PD patients at four time points during a 6-week balance-training program (Study2), collected in two earlier studies, were used. COP was recorded in four tasks, two on a rigid surface and two on foam, both with eyes open and eyes closed. A postural control model (an inverted pendulum with a Proportional-integral-derivative (PID) controller and time delay) was fitted to the COP data to subject-specifically identify the model parameters thereby calculating |*GM*| and PM for each subject in each task.

**Results:** PD patients had a smaller margin of stability (|*GM*| and *PM*) compared with HCs. Particularly, patients, unlike HCs, showed a drastic drop in *PM* on foam. Clinical outcomes and margins of stability improved in patients after balance training. |*GM*| improved early in week 4, followed by a plateau during the rest of the training. In contrast, *PM* improved late (week 6) in a relatively continuous-progression form.

**Conclusion:** Using fundamental stability theorems is a promising technique for the standardized quantification of postural stability in various tasks.

## 1 Introduction

Postural instability is a cardinal sign and a common feature of Parkinson’s disease (PD). Although postural instability occurs in various conditions such as multiple sclerosis, chronic stroke, and even elderly people, it is notable in Parkinson’s disease (Appeadu and Gupta, 2020). It usually presents at diagnosis, but worsens with disease progression and largely manifests in the late stages of the disease (Marchese et al., 2003; Kim et al., 2013). Impaired postural control is a major source of disability and loss of mobility, which predisposes patients to unexpected falls and compromises autonomy and quality of life (Nantel et al., 2012). Postural instability may initially manifest as the inability to recover equilibrium when pushed or tripped; it usually progresses to the dysfunction of salient tasks such as sitting or standing (Horak et al., 1992). Patients are likely to fall when they have to change the position of the center-of-mass (COM), such that the vertical projection of their COM considerably moves within the base of support (BOS) (Frenklach et al., 2009). Falls occur when patients attempt to or actively produce transitional movements (such as sit-to-stand, turning, and walking), as the vertical projection of the COM tends to be at to the outermost boundary of the BOS (Mancini et al., 2008; Frenklach et al., 2009). In dynamic conditions, in which the BOS is dynamically reshaped through time, the vertical projection of the COM even sometimes overtakes the boundaries of the BOS, disposing patients to short periods of mechanical instability, that is then recovered by taking the step strategy (e.g., the next step during walking, or the compensatory step in response to intense perturbations) (Curtze et al., 2010). There is growing evidence that physical therapy and particularly balance exercises can improve postural stability and reduce fall risk (Goodwin et al., 2008; Kim et al., 2013; Abbruzzese et al., 2016).

The exact quantification of postural instability, although quantification has an established clinical implication, is still an open question (Siragy and Nantel, 2018; Olsson et al., 2021). In clinical practice, postural stability is defined through functional and task-based tests, i.e., the ability to maintain equilibrium under both static (e.g., quiet stance) and dynamic conditions (e.g., in response to perturbations or in volitional movements) (Mancini et al., 2008; Kim et al., 2013; Diab et al., 2014). From this perspective, many clinical tests (e.g., the retropulsion- or pull-test, tandem and single-leg stance, and the Timed Up and Go test (TUG)) and rating scales (e.g., the Berg Balance Scale (BBS) and Balance Evaluation Systems Test (BEST)), although being subjective (Mancini and Horak, 2010; Nonnekes et al., 2015), are commonly used to evaluate postural stability (Johnson et al., 2013; Tomlinson et al., 2013). On the other hand, laboratory tests (e.g., quiet stance, dynamic, or moving-platform posturography) are proposed as objective tools to quantify postural stability. Fundamentally, the classic clinical tests have been translated into laboratory form by the advents of state-of-the-art devices, which have further evolved into the current technology-based tests. For instance, the Limit of Stability test (LOS) (Didier et al., 2014) is an advanced form of the former Functional Reach Test (FRT), benefiting from clear-cut metrics.

Despite the advances in the evaluation and quantification of postural stability, current clinical and laboratory tests are less attributed to the biomechanical definition of stability from an engineering viewpoint. In other words, clinical balance tests assess merely a general functionality in limited and specific balance-related activities rather than assessing the fundamental stability theorems that take into account human system dynamics and the generic underlying governing rules (e.g., the gain assigned by the central nervous system (CNS) to the position/velocity information from the sensory systems, the processing time the CNS takes to issue the control commands, the feedback time delay, and the safety margin that the CNS adopts, any of which shares a specific similar mathematical formulation in the biomechanical/neuromechanical modeling of various static and dynamic tasks, although with different values). As a result, the clinical implication of stability, which is rated by clinical balance tests, cannot be directly addressed by experimental tests which mainly focus on postural control biomechanics. To address this issue, computational biomechanical/neuromechanical models should be developed to model/explain clinical tests; as such, clinical and experimental tests will be represented and discussed through unified descriptive terms (e.g., by similar stability criteria from control engineering). As an example, the widely used clinical balance tests such as the FRT or Modified Romberg Test, similar to quiet- (Maurer and Peterka, 2005) or perturbed- (Boonstra et al., 2013) stance tasks, can be studied through computational models, i.e., to develop a biomechanical model (e.g., a single- or double-inverted pendulum) with a high-level controller (which normally has a known common mathematical formulation scheme in control engineering regardless of the task type). The parameters of the controller are dynamically set as the computational model is simulated via its underlying governing mathematical formulations, throughout the time of simulation. Indeed, each specific parameter in the mathematical formulation of the controller, has a specific intuitive meaning, which is unique and shares similar meaning in all types of tasks (e.g., gain assigned by the CNS to the position/velocity information, time-delay, safety margin of stability, and level of stability), which explain the characteristics of the neuromechanical system of the human posture. The different values that emerge in these parameters, further unravel the underlying governing rules of that task or the defects in the balance performance of a specific group of patients. These governing rules, in turn, are set such that to meet all engineering criteria, such as stability criteria, regardless of the task type. In this framework, the system dynamics account for the considered biomechanical model, involved segments, and motions, which can differ from task to task, and the controller scheme normally has a unique, similar formulation and governing rules from control engineering (except for the degree-of-freedom of the controller which is adjusted in accordance to the degree-of-freedom of the involved biomechanics). The broad application of such computational frameworks and modeling in clinical and laboratorial tests allows for the emergence of unified descriptive terms (e.g., the margin or level of stability) in both disciplines, which further enhances the linkage of clinical implications to the laboratorial findings.

Some studies (Hof et al., 2005; Horak et al., 2005; Siragy and Nantel, 2018; Olsson et al., 2021), including our own (Rahmati et al., 2019), have integrated experimental tests with computational methods (either biomechanical/neuromechanical (Hof et al., 2005; Kim et al., 2009; Fujimoto and Chou, 2014; Hof and Curtze, 2016; Rahmati et al., 2019) or analytical models (Horak et al., 2005; Błaszczyk and Orawiec, 2011; D'An et al., 2017; Termoz et al., 2008)) to more accurately and meaningfully quantify postural stability; however, they have still disregarded control stability theorems in their studies. Horak et al. (Horak et al., 2005) suggested the difference between peak center-of-pressure (COP) and peak COM displacement after platform translation as the margin of stability, which has since been used in various other experiments (Frank et al., 2000; Jacobs et al., 2005; de Lima-Pardini et al., 2012). Hof et al. (Hof et al., 2005) proposed the extrapolated COM (*X*_com_) in dynamic tasks, considering the velocity of COM. They suggested that *X*_com_ should remain in the BOS, as to satisfy the dynamic stability criterion; hence, the difference between *X*_com_ and the maximum boundary of BOS determines the margin of stability. This approach also became popular in the quantification and assessment of stability degree in different studies (such as stability during perturbed walking (Martelli et al., 2017), obstacle crossing (Stegemöller et al., 2012), the sit-to-stand task (Fujimoto and Chou, 2014), and compensatory stepping response after perturbation (Peterson et al., 2016)). However, Hof himself and Curtze argued and refined their proposed criteria (Hof et al., 2005) in a later study (Hof and Curtze, 2016), showing the necessity to consider human dynamics and the intrinsic time delay in particular. Nevertheless, Hof et al. (Hof and Curtze, 2016) still relied on the temporal and spatial characteristics of COM, regardless of the inherent ruling stability criteria which are explored in control engineering. In an attempt to examine the robustness of human postural control (another term for evaluating the margin of stability), Hur et al. (Hur et al., 2010) studied a human postural control model using engineering stability criteria, examining the known measures, gain margin (*GM*) and phase margin (*PM*) in control engineering, for discriminating age-related reduced postural stability in healthy subjects; however, they still did not address the clinical implications. To quantify stability, researchers have presented novel quantitative terms such as stability degree (Rahmati et al., 2019), region of stability (Fujimoto and Chou, 2014), limit of stability (Mancini et al., 2008; Eysel-Gosepath et al., 2016), and margin of stability (in quiet stance (Hof et al., 2005) or dynamic states (Horak et al., 2005)) in clinical applications for healthy subjects or PD patients, some of them considering biomechanical models; however, disregarding control stability theorems. The exact quantification of postural stability in clinical applications using biomechanical stability criteria (such as stability criteria in control engineering) provides an accurate interpretation of clinical and laboratory balance tests through unified terms, which can potentially link clinical tests to the realm of experimental studies.

In this study, we investigated the feasibility of a widely used stability criterion from control theory, known as the Nyquist criterion (Ogata, 2010) (i.e., gain margin (*GM*) and phase margin (*PM*)) for quantification of the postural stability in PD. The idea was to discriminate between PD patients and healthy control subjects (HCs), as well as to analyze the adjustments in the patients over time, during the course of a balance-training program. Particularly, the proposed stability terms are determined based on the quiet stance posturographic data (which is low-cost and easily accessible in clinics) and benefiting from a subject-specific computational postural control model. For this purpose, two datasets from our two previous studies (Rahmati et al., 2019; Rahmati et al., 2020), comprising quiet stance posturographic data of PD patients before, during, and after a balance-training program and HCs were used. A postural control model was fitted to the COP data of each subject, and as such identifying each subject’s model parameters. Next, *GM* and *PM* were calculated for each subject (HC or PD before, during, and after training) using the subject-specific identified model. Inspecting the *GM* and *PM* for HCs versus PD patients, as well as the reflection of adjustments in postural stability of PD patients during balance training in these new terms, presents a new quantification tool for postural stability in PD, i.e., the margin of stability.

## 2 Materials and methods

### 2.1 Participants and experimental procedures

The data were taken from our two previous studies (Rahmati et al., 2019; Rahmati et al., 2020). As such, the materials come summarily here, and the reader is referred to those articles for more details.

*Study1* (Rahmati et al., 2019):

Forty PD patients (seven female, 63.1 ± 12.1 years, Hoehn-Yahr ≤3, Mini-Mental State Examination (MMSE) score ≥24) and 20 healthy age-, height-, and weight-matched control subjects (four female, 63.8 ± 12.1 years) participated in the study. The patients were assessed clinically and experimentally, before and after a 4-week (12-session) balance-training program. Patients attended training sessions every non-consecutive day (3 days/week) for 4 weeks. Training sessions included 45–60 mins balance exercises with an extra 30 mins of conventional rehabilitation, based on the task difficulty and safety of patients. Balance exercises included maintaining balance in different conditions (e.g., quiet standing, tandem standing, and semi-tandem standing) while receiving the following types of sensory stimulation: 1) proprioceptive stimulation, 2) visual stimulation (tracking different images and videos displayed on a monitor in front of the patients), 3) vestibular stimulation (using a balance board and different movements of the head), and 4) the combination of visual, vestibular, and proprioceptive stimulations. The experimental assessments included force-plate quiet stance posturography (the recording of COP data at 1 kHz for 70 s) in eight trials) (four sensory tasks, two trials in each): quiet stance on a rigid surface with eyes open (RO) and closed (RC), and quiet stance on foam (10.5 × 40 × 60 cm, 20 Kg/m^3^ density, and 4,000 N/m^2^ elastic modulus) with eyes open (FO) and closed (FC). HCs were assessed by experimental tests, and only once. Participants were instructed to stay quiet with their arms close to their body in all four conditions; the trial was repeated in the case of any considerable arm or trunk movements.

*Study2* (Rahmati et al., 2020):

Twenty other PD patients (five female, 63.3 ± 7.5 years, Hoehn-Yahr ≤3, MMSE ≥24) participated in the study. Patients were assessed clinically and experimentally before, during, and after a 6-week (18-session) balance-training program. Training sessions were held 3 days per week for 6 weeks. Each session included a 10-min warm-up followed by 20 mins of conventional rehabilitation (such as stretching, range-of-motion exercise, body-weight strengthening of hip and ankle, volitional/large stepping, and forward/backward/sideways walking), and 30–60 mins of balance exercises. The balance exercises included both overground balance exercises and device-based exercises, performed by the laboratory-developed device, *Balance Robot*. The exercises with the *Balance Robot* included Limit of Stability (LOS), Random Control, and Postural Stability (Rahmati et al., 2020). The exercises’ difficulty levels and their challenges were designed progressively throughout sessions to maintain the engagement of patients with the training program. Particularly, the platform beneath the patients was subject to disturbance levels 0, 1, and 2 (as described in detail in Rahmati et al. (2020)) during the weeks of 0–2, 2-4, and 4–6 weeks, respectively. Experimental assessments were similar to *Study*1, i.e., the collection of COP data with a similar procedure: in four tasks (RO, RC, FO, and FC), each with two trials. As for capturing the patterns of changes in patients during the training program, experimental tests were taken at four time points: before (Pre, or week0), at week 2 (week2), at week 4 (week4), and after training (Post, or week6).

All PD patients in both *Study*1 and *Study*2 were diagnosed based on the UK Parkinson’s Disease Society Brain Bank criteria (Gelb et al., 1999) and had no other comorbidities (e.g., neurological, orthopedic, or musculoskeletal disorders). The entire assessment and training sessions in both studies were held while patients were ON-medicated, i.e., 1–2 h(s) after taking their normal medication. Furthermore, the order of four experimental tasks was randomized for each subject to avoid any bias caused by learning effects. Subjects were allowed to have sufficient rest intervals between trials if they needed. All participants provided written confirmed consent, in compliance with the Declaration of Helsinki. The local ethics committee approved both studies.

### 2.2 Data analysis

COP data of subjects in both studies were first used to identify the parameters of a subject-specific postural control model as described fully in Rahmati et al. (2019), and briefly in the following. Next, the variables of the Nyquist stability criterion, i.e., the gain margin (*GM*) and phase margin (*PM*) for each subject in each task and at each time point of assessment were calculated using the identified subject-specific model, as given below.

#### 2.2.1 COP analysis and model description

**FIGURE 1:**
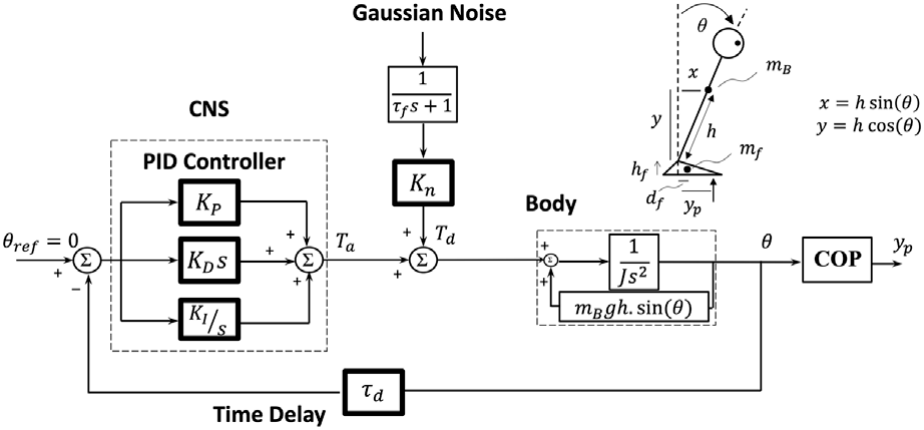
The subject-specific postural control model. The model consisted of a human ‘Body’, CNS in the form of a PID controller, and time delay (*τ*_*d*_). The ‘Body’ was modeled by an inverted pendulum with all mass (*m*_*B*_) centered at the height of the *COM (h)* (which were adjusted subject-specifically). *J* is the moment of inertia of the body around the ankle axis. The COP displacement (*y*_*p*_) was calculated from the body sway angle (*θ*) considering the feet mass (*m*_*f*_ = 2.01 kg), which is fully described in Rahmati et al. (2019). The PID controller represents the CNS control performance: *K*_*P*_ (proportional gain), *K*_*D*_ (derivative gain), *K*_*I*_ (integral gain); *s*, the Laplace transform variable (the frequency-domain); *T*_*a*_, corrective ankle torque; *T*_*d*_, disturbance torque; *K*_*n*_, internal disturbance torque gain which quantifies the flexibility degree; *τ*_*f*_ = 100 s, time constant for the low-pass filter.

COP data were filtered (10 Hz, 3rd order Butterworth) and resampled to 100 Hz. From the COP data, the parameters of a subject-specific postural control model (Figure 1) (consisting of an inverted pendulum—with mass and length adjusted to the corresponding subject—as the body part and a proportional-integral-derivative controller with time delay representing the central nervous system (CNS)) were identified. The parameters, including PID controller gains (i.e., *K*_*P*_, *K*_*D*_, and *K*_*I*_), time delay (*τ*_d_), and the disturbance torque gain (also called noise gain—*K*_*n*_) were estimated through an optimization algorithm, minimizing the difference between the simulated and the experimentally extracted COP-based sway measures (Rahmati et al., 2019). Since all the experimental tasks were performed using only the ankle strategy (without employing the hip or step strategy in balance maintenance), a single inverted pendulum was regarded in the study. In numerous previous studies, the inverted-pendulum model was shown as a promising model to soundly explain the biomechanical/neuromechanical behavior of the human postural control in various tasks in healthy (Winter et al., 1997; Maurer and Peterka, 2005; Wiesmeier et al., 2017) and PD patients (Boonstra et al., 2014; Petrucci et al., 2018; Rahmati et al., 2019). Chiba et al. (Chiba et al., 2016) and Olsson et al. (Olsson et al., 2021) extensively reviewed the biomechanical and neuromuscular control models which were investigated to explain the human postural control characteristics.

#### 2.2.2 Nyquist stability criterion measures (gain margin and phase margin)

The Nyquist stability criterion can be applied to any linear system as long as it is presented by a frequency-response function (FRF) or transfer function. By this criterion, the stability analysis of a system reduces to some specific mathematical conditions in the frequency domain. In this regard, gain margin (*GM*) and phase margin (*PM*) are calculated from the system FRF (Supplementary Appendix). To meet the stability criteria, both *GM* and *PM* should be positive, unless the system is a non-minimum phase system (as is the case of our non-minimum phase system, which has two *GM*s with a negative and a positive sign, yet remains stable) (Ogata, 2010). In this case, the alternative Nyquist stability criterion (the Nyquist plot) examines the stability of a system and accepts or rejects the negative sign of *GM*/*PM* while remaining stable. The Nyquist stability criterion indicates not only whether a system is stable but also the degree of stability of a system in terms of the parameters *GM* and *PM* (Ogata, 2010). *GM* indicates how much the controller gain of a system can be increased/decreased before the system becomes unstable. *PM* is the amount of additional phase lag (e.g., time delay) required to bring the system to the verge of instability (Ogata, 2010). As much as the values of *GM* and *PM* for a system be closer to the instability border (closer to zero), the safety margin for the stability of the system is reduced. In this sense, *GM* and *PM* imply the safety margin for controller adjustments (in gains and time delay) before making the system unstable; or simply, the margin of stability in the human stance.

The subject-specific estimated model was rearranged into a transfer function (Supplementary Equation A3) (considering the linearized inverted pendulum around the upright position – Supplementary Equation A1), from which the *GM* and *PM* were calculated (Supplementary Equations A4–A7). For a detailed calculation of *GM* and *PM*, refer to the Supplementary Equations A4–A7. We used the MATLAB v.8.1 (Mathworks Inc., MA, USA) function ‘*margin*’ to calculate the *GM* and *PM* for each subject, in each task, and at each time point of assessment. Furthermore, the stability of the estimated system for each subject, task, and time-point was examined by the Nyquist criterion (by the Nyquist plot). Typically, all estimated systems were stable, given that subjects performed all tasks stably. Besides the stability check, we were interested in the quantification and the degree of such stability; therefore, values of *GM* and *PM* were analyzed as well. The Nyquist stability criterion showed that both negative and positive *GM* satisfy the stability of the inverted-pendulum system, providing that *PM* is positive. Therefore, from two values of *GM*s for each subject in each task, we chose the minimum absolute value (denoted by |*GM*|) and the exact value of *PM*, respectively, as the measures of margin of stability (“stability margin measures”).

### 2.3 Statistical analysis

The normal distribution of |*GM*| and *PM* was tested by the Shapiro–Wilk normality test. All stability margin measures (|*GM*| and *PM*) were normally distributed.

*Study1*: Differences between the margins of stability (|*GM*| and *PM*) of PD patients at baseline before training (PD-Pre) and HCs were tested by a 2 × 2 × 2 mixed model analysis of variance (ANOVA). The mixed model ANOVA had two groups (PD and HC) as the between-subjects factors, and two visual levels (eyes open (EO) and eyes closed (EC)) and two surface conditions (rigid (R) and foam (F)) as the within-subjects factors. The post-hoc multiple comparisons were carried out using Bonferroni correction. To evaluate the changes in the stability margin measures of patients before (PD-Pre) and after training (PD-Post), the paired sample t-test was used.

*Study2*: The temporal changes (the pattern of improvement) in stability margin measures of PD patients during the balance-training program were evaluated using repeated measures ANOVA with one factor (*Time*) in each individual task. The factor *Time* comprised four levels for the stability margin measures (Pre, week2, week4, and Post). The Bonferroni-corrected post-hoc multiple comparisons evaluated differences between time points.

The significance level in both studies was set at 0.05.

## 3 Results

### 3.1 Study1; margin of stability in PD patients vs. healthy controls

Table 1 summarizes the values of |*GM*| and *PM* for 20 healthy control subjects (HCs) and 40 PD patients before and after the 4-week balance-training program in four tasks.

**TABLE 1:** (*Study*1) Stability margin measures (absolute value of gain margin |*GM*| and exact value of phase margin *PM*) for 20 healthy control subjects, as well as 40 patients with PD before and after a 4-week balance-training program in four tasks: stance on rigid surface with eyes open (RO) and closed (RC) and stance on foam with eyes open (FO) and closed (FC). Values of *GM* are in decibels (dB), which is 20*Log_10_(.) of the gain margin value.

Measures of margin of stability	Task											
	Healthy control subjects (n = 20)				PD—Pre training (n = 40)				PD—Post training (n = 40)			
	RO	RC	FO	FC	RO	RC	FO	FC	RO	RC	FO	FC
|*GM*| (*dB*)	3.74 ± 1.64	4.31 ± 1.97	3.49 ± 1.17	4.72 ± 1.37	2.99 ± 1.15*	3.15 ± 1.43*	2.60 ± 0.8**	3.05 ± 1.12**	3.42 ± 1.38	3.34 ± 1.54	3.24 ± 1.43‡	3.63 ± 1.2†
*PM* (*deg*)	15.47 ± 5.6	13.47 ± 5.3	15.21 ± 7.3	16.22 ± 6.8	15.84 ± 6.1	15.21 ± 5.59	13.85 ± 5.7	13.43 ± 6.14	16.67 ± 8.0	15.28 ± 5.95	18.92 ± 7.6‡	15.24 ± 6.7

Significant difference between Healthy control subjects and PD-Pre, independent *t*-test: *p < 0.05, **p < 0.013.

Significant difference between PD-Pre and PD-Post, paired sample *t*-test: †*p* < 0.05, ‡*p* < 0.013.

**FIGURE 2:**
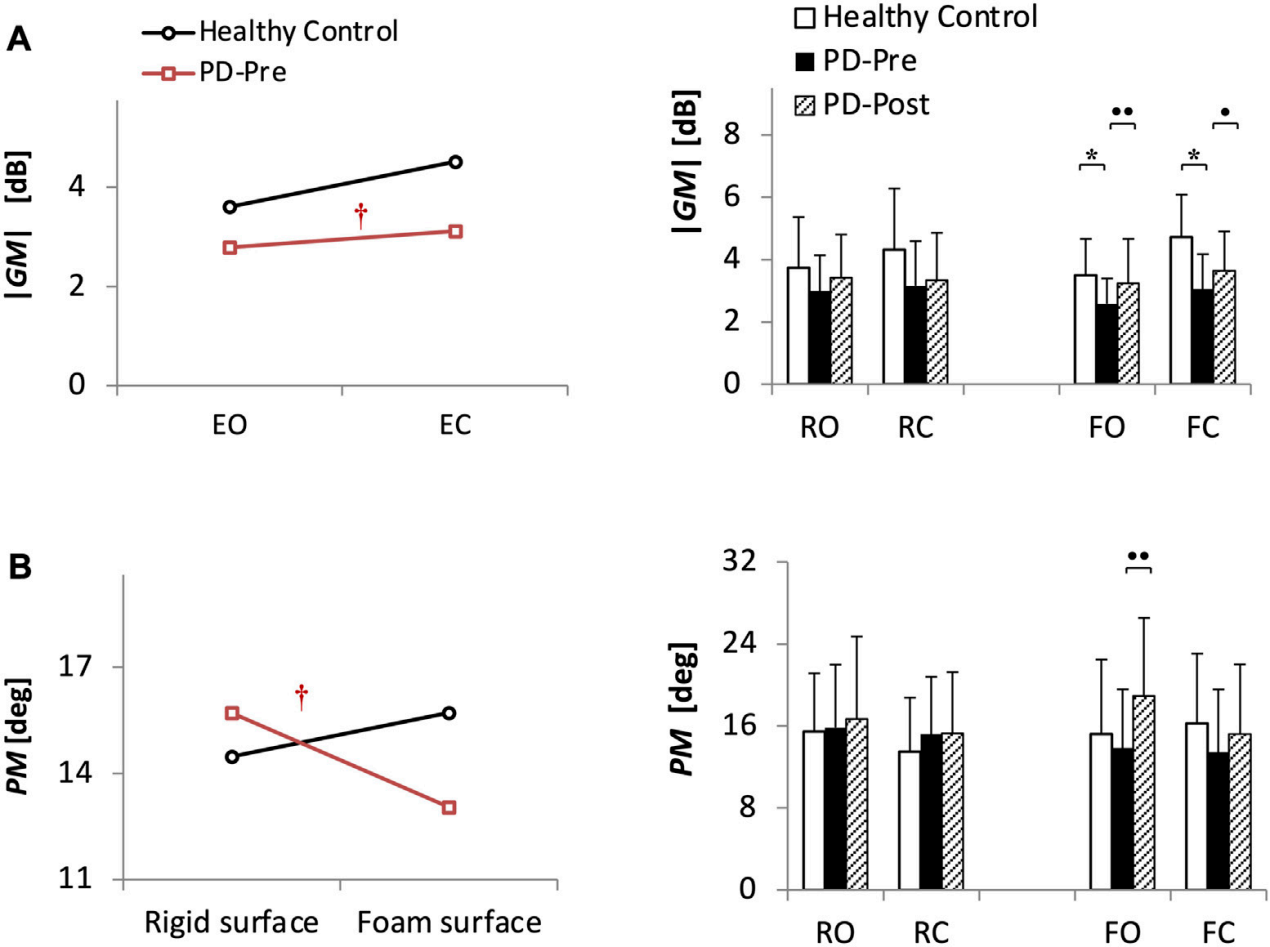
(*Study*1) Stability margin measures (**(A)** absolute value of gain margin |*GM*| and **(B)** exact value of phase margin *PM*) for 20 healthy control subjects (HCs) and 40 patients with PD before (PD-Pre) and after (PD-Post) a 4-week balance-training program. Profile plots show the significant results from ANOVA analysis comparing HCs and PD-Pre. †: Significant interaction (*p* < 0.05). Bar charts present the results of Bonferroni post-hoc comparisons between HCs and PD-Pre, * (*p* < 0.05), as well as paired sample *t*-test results between PD-Pre and PD-Post, • (*p* < 0.05), •• (*p* < 0.013). Values of *GM* are in decibels (dB), which is 20*Log_10_(.) of the gain margin value. RO, stance on rigid surface with eyes open; RC, stance on rigid surface with eyes closed; FO, stance on foam with eyes open; FC, stance on foam with eyes closed.

*PD-Pre vs. HCs*:

Figure 2 and Table 2 present the ANOVA results comparing 40 PD patients before balance training (PD-Pre) with 20 HCs. Patients had a smaller |*GM*| compared with HCs (Table 2, group effect: *p* = 0.0001), particularly in the tasks on foam (F-tasks) (Figure 2A, FO: *p* = 0.008; FC: *p* = 0.00005). Furthermore, patients exhibited a relatively smaller |*GM*| than HCs with closing eyes (Table 2, group × vision: *p* = 0.039). As for the *PM*, the group × surface effect (Table 2, *p* = 0.008) showed that standing on foam resulted in a drastic drop in the *PM* of PD patients, whereas HCs preserved the same level of *PM* on foam as that on the rigid surface (Figure 2B).

**TABLE 2:** (*Study*1) The results of the ANOVA analysis comparing the measures of margin of stability (absolute value of gain margin |*GM*| and the exact value of phase margin *PM*) between Healthy Controls (HCs) and the PD patients before balance training (PD-Pre).

ANOVA (healthy controls, PD-Pre)—*p*-value (*F*-value)		
Factor	Measures of margin of stability	
	|*GM*|	*PM*
Group	**0.0001 (17.7)**	0.621 (0.247)
Vision	**0.00003 (21.1)**	0.635 (0.229)
Surface	0.573 (0.322)	0.440 (0.607)
Group × Vision	**0.039 (4.5)**	0.948 (0.004)
Group × Surface	0.305 (1.1)	**0.008 (7.7)**

Significant *p*-values are in bold.

*Effects of the 4-week balance training on 40 PD patients*:

Balance training increased the |*GM*| and *PM* of patients in F-tasks (Figure 2, |*GM*|: FO: *p* = 0.006, FC: *p* = 0.033; *PM*: FO: *p* = 0.00048).

### 3.2 Study2; patterns of improvement in the margin of stability in PD during balance training

**FIGURE 3:**
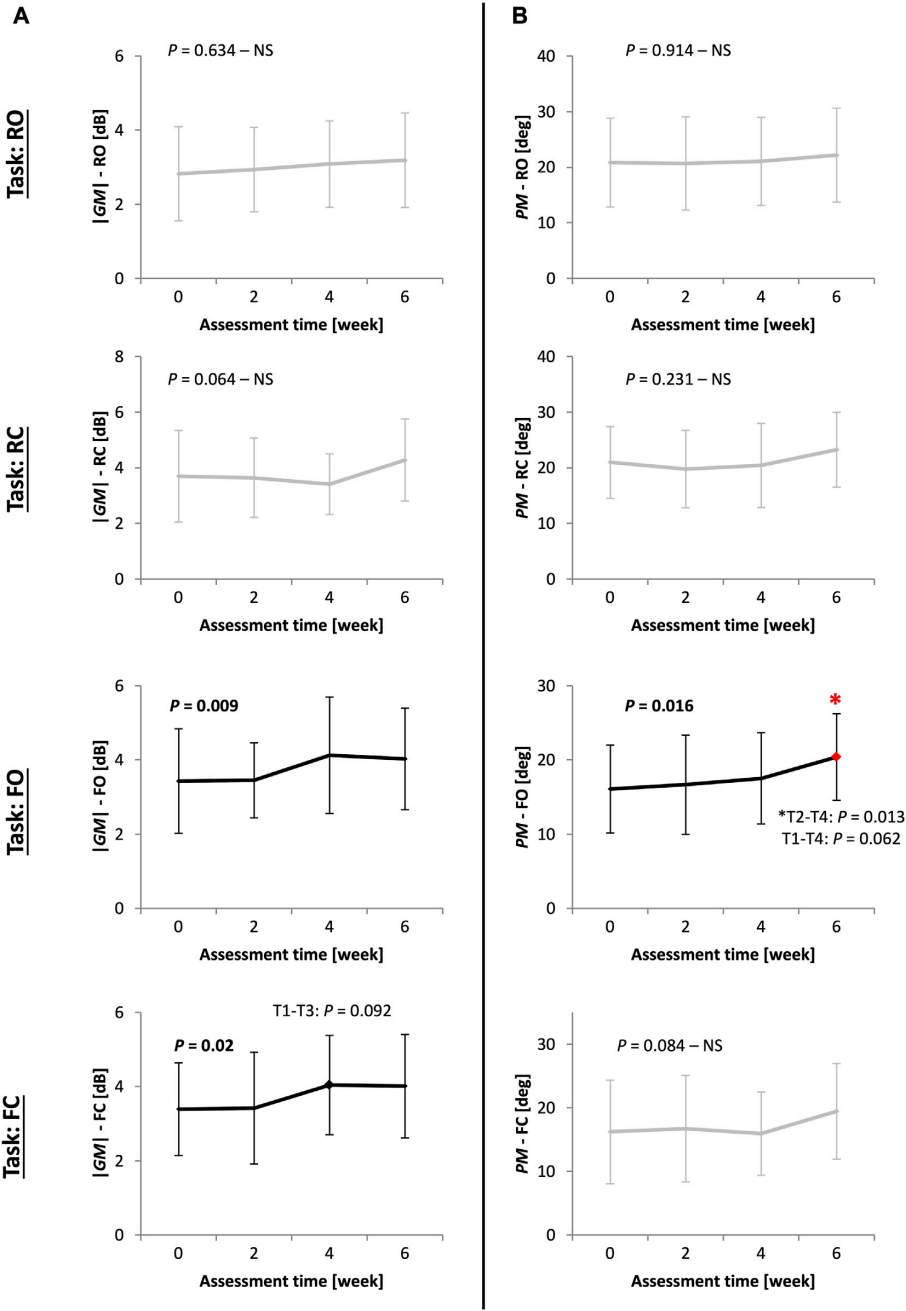
(*Study*2) The pattern of improvement for stability margin measures (**(A)** absolute value of gain margin |*GM*| and **(B)** exact value of phase margin *PM*) for 20 other PD patients in *Study*2 at four time points (Pre-, week2, week4, and Post-training) during a 6-week balance-training program in four tasks: stance on rigid surface with eyes open (RO) and closed (RC); stance on foam with eyes open (FO) and closed (FC). ANOVA results showing significant improvements are in bold black lines. Bonferroni *p*-values are reported for post-hoc pairwise comparisons between time points, with the asterisk showing significant differences (T1-T4 stands for Pre-, week2, week4, and Post-training assessments). Values of *GM* are in decibels (dB), which is 20*Log_10_(.) of the gain margin value.

Table 3 and Figure 3 present the values and statistical results of the stability margin measures (|*GM*| and *PM*) for the other 20 PD patients in *Study*2 during the 6-week balance-training program at four time points: at Pre-, week2, week4, and Post-training. The margin of stability improved specifically in F-tasks (significant improvements are depicted with bold black line curves).

**TABLE 3:** (*Study*2) Stability margin measures (absolute value of gain margin |*GM*| and the exact value of phase margin *PM*) for 20 other PD patients in *Study*2 during 6-week balance-training program at four time points (at Pre-, week2, week4, and Post-training), and in four tasks (stance on rigid surface with eyes open (RO) and closed (RC), and stance on foam with eyes open (FO) and closed (FC)). Values of *GM* are in decibels (dB), which is 20*Log_10_(.) of the gain margin value.

Task	PD patients (n = 20)				ANOVA *p*-value (*F*-value)	Effect size	Bonferroni *p*-value for post-hoc comparisons					
Measures of margin of stability	Pre (*T*1)	week 2 (*T*2)	week 4 (*T*3)	Post (*T*4)			*T*1-*T*2	*T*1-*T*3	*T*1-*T*4	*T*2-*T*3	*T*2-*T*4	*T*3-*T*4
RO												
|*GM*| (*dB*)	2.82 ± 1.27	2.94 ± 1.14	3.08 ± 1.16	3.19 ± 1.27	0.634 (0.574)	0.029	1.000	1.000	1.000	1.000	1.000	1.000
*PM* (*deg*)	20.85 ± 7.98	20.68 ± 8.38	21.04 ± 7.90	22.18 ± 8.44	0.914 (0.173)	0.009	1.000	1.000	1.000	1.000	1.000	1.000
RC												
|*GM*| (*dB*)	3.69 ± 1.65	3.64 ± 1.43	3.41 ± 1.09	4.28 ± 1.47	0.064 (2.55)	0.118	1.000	1.000	0.735	1.000	0.253	0.081
*PM* (*deg*)	20.95 ± 6.45	19.75 ± 6.96	20.40 ± 7.57	23.24 ± 6.72	0.231 (1.474)	0.072	1.000	1.000	0.951	1.000	0.327	0.682
FO												
|*GM*| (*dB*)	3.43 ± 1.41	3.45 ± 1.01	4.13 ± 1.57	4.03 ± 1.37	**0.009** (4.193)	0.181	1.000	0.149	0.173	0.278	0.292	1.000
*PM* (*deg*)	16.08 ± 5.92	16.65 ± 6.68	17.52 ± 6.17	20.39 ± 5.84^a^	**0.016** (3.736)	0.164	1.000	1.000	0.062	1.000	**0.013**	0.158
FC												
|*GM*| (*dB*)	3.39 ± 1.25	3.42 ± 1.51	4.04 ± 1.34	4.01 ± 1.39	**0.020** (3.55)	0.157	1.000	0.092	0.164	0.279	0.289	1.000
*PM* (*deg*)	16.21 ± 8.15	16.72 ± 8.37	15.94 ± 6.55	19.45 ± 7.52	0.084 (2.325)	0.109	1.000	1.000	0.510	1.000	0.526	0.075

Values are reported as mean ± standard deviation. Significant *p*-values are in bold.

*T*1 to *T*4 refer to Pre-, week 2, week 4, and Post-training, respectively.

^a^ Significantly different from week 2 (*p* < 0.05).

|*GM*| improved in F-tasks (FO: *p* = 0.009, FC: *p* = 0.02). Improvement in |*GM*| was characterized by an early improvement at week 4 followed by a plateau over the next two final weeks of training (Figure 3A). Unlike |*GM*|, *PM* improved late, at week 6 (*T*4 to *T*2 comparison: *p* = 0.013), in an approximately gradual and continuous-progression form (Figure 3B).

## 4 Discussion

The stability measures |*GM*| and *PM* from control theory were novelly used in this clinical study to quantify the stability degree in patients with Parkinson’s disease, as well as to evaluate the effects of balance training in PD. The pattern of improvements during a 6-week balance-training program, in terms of gain and phase margin, was investigated in PD patients. |*GM*| and *PM* were calculated using the low-cost posturographic data and a computational subject-specific postural control model. Findings showed that the stability safety margins (i.e., |*GM*| and *PM*) were smaller in patients compared with healthy control subjects (HCs). Patients, unlike HCs, significantly reduced *PM* on foam, which is known to be due to a considerable time delay from an engineering viewpoint. Stability margins improved in patients after balance training. Improvement in |*GM*| was characterized by an early improvement at week 4 followed by a plateau during the next two final weeks of training. In contrast, *PM* improved relatively late at week 6 in a rather continuous-progression form.

### 4.1 PD patients adopting smaller margin of stability: the clinical implications

A reduced gain margin (|*GM*|) in PD, as we observed in *Study*1, is in line with previous studies which reported a lower margin of stability for PD patients compared with HCs in different tasks (e.g., perturbed quiet stance (Horak et al., 2005; Jacobs et al., 2005) or perturbed gait (Martelli et al., 2017)). However, considering the different tasks and techniques of quantification in those studies, any inference should be drawn cautiously. Although most of these studies employed the spatial term of the margin of stability (i.e., the difference between peak COM and peak COP), Patton et al. (Patton et al., 1999) showed that the torque safety margin (which is mainly addressed in our study by considering the corrective ankle torque in our model) is highly correlated with the spatial safety margin calculated from COP response characteristics. From the engineering viewpoint, |*GM*| is substantially influenced by the value of controller gain parameters in a system, which in turn is mainly associated with the produced ankle torque or the muscular strength of the human body, suggesting that the reduced |*GM*| in PD, in the framework of our neuromechanical model, may be due to weakened muscle strength (Carpenter et al., 2004; Boonstra et al., 2014). Recently, we showed that most of the control parameters (such as *K*_*P*_, the pivotal ruling gain parameter) were lower in PD compared with HCs (Rahmati et al., 2019). Nevertheless, an excessive increase in gain parameters results in resonant instability (Asai et al., 2009). In other words, it is well accepted in control engineering that *K*_*P*_ should remain between a lower and an upper bound to guarantee the stability of the inverted-pendulum system. This implies that, in fact, the CNS tunes all control gain parameters in a way to adjust the margin of stability; that is, the resultant |*GM*| in our neuromechanical model is truly expressing the margin of stability rather than the muscular strength (*K*_*P*_). That is, |*GM*| carried a different implication of stability from *K*_*P*_ (the other measure for quantification of stability degree) and potentially can be reflected in more dynamic challenging tasks in future studies. In a similar study considering *GM* and *PM* on healthy subjects and the task of response to perturbation, Hur et al. (Hur et al., 2010) also observed that only some control parameters were correlated to their proposed ‘robustness measure’ (another term for defining the margin of stability in control theory). Interestingly, control parameters failed to discriminate age-related reduced margin of postural stability, while the proposed robustness measure showed a significant difference in the safety margin (robustness) that the CNS adopts between young and elderly groups, favoring the fact that |*GM*| is presenting its unique essence of safety margin as is regarded in engineering viewpoint. Furthermore, our results showed that HCs and patients increased |*GM*| when closing their eyes, supporting the impression that the CNS adopted a higher level of safety margin in more threatening and challenging tasks. An extended safety margin in EC tasks was seen in young (Błaszczyk et al., 2020) and healthy elderly subjects (Blaszczyk et al., 1994), as well as PD (Stegemöller et al., 2012) and multiple sclerosis patients (Craig et al., 2019). The group by vision interaction by vision interaction in our study, however, revealed that PD patients, although they supposed to take a higher level of safety margin due to the inherent fear of fall they have, did not adjust (augment) |*GM*| as much as HCs did in EC tasks, reiterating the contribution of the reduced strength factor in patients, as evidenced by the low control gain *K*_*P*_ in EC tasks in our recent study (Rahmati et al., 2019).

Unlike |*GM*|, the *PM* was almost similar in PD patients and HCs, although with a drastic drop for PD patients on foam. It seemed plausible, in that all subjects (patients or HCs) performed all tasks stably, which denotes (from the engineering viewpoint) that both groups of patients and HCs should a moderately similar *PM*, although having different |*GM*|. Stability in a delayed-inverted-pendulum system relies largely on the adequacy of time delay, which should not violate a critical value (Landry et al., 2005; Molnar et al., 2020). It is well accepted in control engineering that time delay, in part, has a remarkable contribution to the amount of *PM*. Therefore, in stable performance, *PM* almost remains in a specific range, unless the time delay varies significantly. Our findings showed that patients, contrary to HCs, exhibited a drastic drop in *PM* while standing on foam (group × surface interaction), indicating the patients’ deficit in preserving the level of *PM* on foam similar to that on a rigid surface. The decline in *PM* for patients on foam, in the framework of this neuromechanical model, may be a consequence of the significant delayed response (longer time delay) that patients had on foam (Rahmati et al., 2019), which brought patients to the verge of instability (as indicated by the smaller *PM* in control engineering). Wright et al. (Wright and Laing, 2011) observed a reduced margin of stability on a compliant surface for a group of elderly healthy women, while MacLellan et al. reported an increased margin of stability for stepping onto and walking on foam for young adults (MacLellan and Patla, 2006), emphasizing the different manner in HCs and elderly (or patient) groups in adjusting *PM* per changes in surface compliance (group × surface interaction). The increase in *PM* on foam for HCs and the decrease in that for PD patients can be seen in the average values in our results. Still, different tasks, or definitions for the margin of stability, may give rise to different possible results, which further highlights the importance of applying fundamental concepts from engineering such as the Nyquist stability criterion for the quantification of stability (or margin of stability) in future studies. Foam posturography is usually believed to be a source of sensory perturbation to the postural control; nonetheless, some researchers showed that standing on foam is not equivalent to the reduction of mechanoreceptive sensation (Patel et al., 2011). Our findings suggest that standing on foam causes a source of longer time delay in the control loop, which can stem from either the CNS processing time or the feedback time delay from the sensory organs (e.g., somatosensory) or both. However, our model is merely based on the biomechanical/physical and neuromechanical attributes of the postural control, and future studies can employ the sensory integration model (Peterka, 2002) to examine the detailed effects of sensory perturbation to the postural control, thereby differentiating the source of the observed time delay (differentiating the CNS processing time from the sensory feedback time-delay). It should be emphasized that the correct sway angle is assumed as available to the CNS in our model, thanks to the redundant sensory systems which provide the correct information of the body sway angle. Moreover, our results imply that the CNS alters the control parameters in accordance with the compliance of the surface (Sozzi et al., 2022). In other words, the foam posturography should not only be interpreted as the perturbation to the sensory system as evidenced in Patel et al. (2011), rather it should be regarded as the context of an altered-compliance environment for the CNS. Our findings indicate that the CNS tries to compensate for this alteration in the compliance of the contact surface by fine tuning its control parameters, which leads to a more conservative or preserved constant safety margin. The need for an altered tuning and adaptive control for the stability of the robotic manipulators in physical human–robot interaction is well documented and observed in control engineering (Ajoudani et al., 2018).

With the multi-modal framework of human postural control (Horak, 2006) in mind, which proposes different factors that contribute to human postural control (such as the influence of cognitive processing, movement strategies, CNS programming and decision-making, and sensory organization) we should be cautious about the interpretations made from the stability safety measures *GM* and *PM*. In fact, *GM* and *PM* in the framework of the proposed neuromechanical model are exclusively capturing the biomechanical/physical factors of human postural control, which directs us to the distinct features of muscular strength (through the produced corrective ankle torque) and neuromechanical delay. However, in studies comprising cognitive loads or tasks with different movement strategies (e.g., anticipatory postural adjustments and sensory organization in PD (Feller et al., 2019)), the contributions from other factors of postural control in the interpretation of the calculated safety margin (*GM* and *PM*) can be distinctly revealed.

### 4.2 Patterns of improvement in the margin of stability during balance training: recommendation for clinical practices

The tracking of stability margin measures (|*GM*| and *PM*) in multiple time points during a course of balance-training program in *Study*2 disclosed their pattern of improvement, enlightening how balance training can affect stability in PD. In addition, the findings suggest that the improvement during training programs can be captured by |*GM*| and *PM* as meaningful and sensitive measures for the assessment of stability in PD using engineering stability criteria and biomechanical/neuromechanical modeling for different tasks. The pattern of improvement in |*GM*| was characterized by an early improvement at week 4, followed by a plateau during the two final weeks. In our recent study (Rahmati et al., 2020), we found similar early improvement with plateaued behavior for stability-related measures such as control gain *K*_*P*_, which we concluded, based on evidence from other studies (Corcos et al., 2013; Roeder et al., 2015; Peterson et al., 2016), as the limited capacity for the development of strength in PD. Although PD patients demonstrated the ability to learn, limited learning capacity in PD has been noted numerously in studies (Abbruzzese et al., 2016; Olson et al., 2019). Corcos et al. (Corcos et al., 2013) noted that 2-year progressive resistance exercise (PRE) in PD, at best, can lead to such plateaued behavior (stagnation at a specific level) for elbow flexion torque in comparison to non-progressive exercise, which progressed during the first months of training and regressed back during the rest of training. Second, some studies have shown early strength gain during the first weeks of training in healthy elderly (Penzer et al., 2015) or PD patients (Falvo et al., 2008; Roeder et al., 2015). These findings indicate that, as an underlying rule, the capability of patients for retaining or improving an adequate margin of stability highly pertains to the capacity of regaining strength. Therefore, |*GM*|, which in the framework of our neuromechanical model is *per se* a reflection of properly tuned gain parameters, improved initially through training but was later subject to plateau after week 4. Possibly, attaining greater values of |*GM*|, such as to the level of more skilled individuals, demands further focused training, particularly in PD. The other stability margin measure, *PM*, showed rather a continuous-progression form that improved late at the end of training in week 6. Our recent study (Rahmati et al., 2020) showed that time delay in PD improved with similar late and continuous-progression form, specifically in the FO task (the very task in which *PM* was improved). Probably, with suggestions from control engineering, improvement in *PM* highly demands the correct timing of control commands in postural control. Such observed patterns in improvements of |*GM*| and *PM* recommend a special focus on strength training during ending sessions and a serious focus on exercises that improve response time during the whole period of the training program (e.g., to include exercises that work on the reduction in reaction time, such as games/tasks with a trade-off in accuracy/precision versus performance speed). A possible explanation for the observed behaviors may be the insufficiency of the challenges and stimuli provided in the exercises, which was extensively discussed in our previous study (Rahmati et al., 2020). However, this is less possible, as we employed progressive difficulty levels for the exercises throughout sessions. Moreover, there is a possibility regarding the content of the balance-training program to have more focus on muscular strength or reaction time in specific intervals of the sessions. Nevertheless, the type of exercises in all sessions was similar, and only the difficulty level (such as the reaching distance or the platform disturbance beneath the patients) was changed throughout the sessions. In addition, the overall content of the training program was based on general balance training and not conventional strength training. At the same time, future studies can assign a ‘strength-focus’ or ‘timing-focus’ score for each exercise to keep the track of the sessions’ training focus in their evaluations. Taken all together, the findings of *Study*2 emphasized that the poor margin of stability in PD is amendable via balance training; nonetheless, specific attention to the necessary dosage at each interval of a training program is required.

A few studies have considered the changes in stability, in terms of the margin of stability, during a training program (Patton et al., 2000; Peterson et al., 2016; Martelli et al., 2017). These studies reported improvements in the margin of stability throughout repeating trials for young (Patton et al., 2000), healthy elderly (Martelli et al., 2017), and PD patients (Peterson et al., 2016; Martelli et al., 2017), although with different test protocols or definitions for margin of stability; nonetheless, they had intriguing results. Given that the spatial margin had a high correlation to the torque safety margin (Patton et al., 1999) (which is associated with gain parameters and therefore |*GM*| as is addressed in our study) facilitates comparing our results with the results from studies with different definition of margin of stability. Peterson et al. (Peterson et al., 2016) realized that the margin of stability (measured as the difference between *X*_com_ and first stepping footfall in response to perturbation) improved in HCs continuously throughout trials, whereas PD patients improved the margin of stability primarily in the first blocks of trials and then plateaued. Patton et al. (Patton et al., 2000) studied the improvement in relative stability while learning a dynamic task (pulling a handle) in ten young subjects. They found that both spatial (the distance-to-boundary for COP to either heel or toe) and temporal (time-to-boundary for COP, using first order predictive extrapolation based on COP velocity) safety margins increased with practice; however, progress in spatial margin was more significant than in temporal margin. Interestingly, Patton et al. observed that spatial margin (for trials with different pulling forces) finally converged into a roughly similar specific value after 5 days of practice, suggesting that, in normal performances or due to biomechanical constraints, only a specific level of spatial margin (similar to *GM*) is achievable. Furthermore, this study revealed that improvement in the temporal margin is hardly achieved via practice, which is in favor of our finding regarding the late and continuous progression of *PM*. This might not be very surprising, since the *GM* and *PM* carry the same nature/essence as the spatial and temporal margin, respectively, in that the *GM* reflects the extent of the torque safety margin (which *per se* predominantly modulates the spatial margin) and the *PM* is mainly attributed to the proper timing in the control loop (as might be reflected in the temporal margin). This might additionally suggest the very fundamental point about the mathematical formulations/framework that is used in control engineering (and therefore in this neuromechanical modeling) to describe the control governing rules (as in the Nyquist stability criteria); i.e. the mathematical formulations of controllers are constructed regardless of the task or the movement type. In other words, in any type of biomechanical/neuromechanical modeling, the whole control rules are simply reduced to the very *GM* and *PM* measures, disregarding the type of task/motion. However, this consistency should be thoroughly examined in future studies.

### 4.3 Limitations and future direction

This study had limitations. First, because of the first-ever usage of *GM* and *PM* for quantification of stability in a clinical application and a neurological disease (PD), the lack of similar studies and evidence limited a more in-depth interpretation of findings, particularly the results on the learning dynamics during a course of the training program. In the most relevant study, Hur et al. (Hur et al., 2010) applied control theory to the postural control of young and elderly healthy subjects in an experimental perturbation task without clinical practice. Previously, spatial (distance-to-boundary) and temporal (time-to-boundary) margins of stability were proposed (Hof et al., 2005) and employed (D'An et al., 2017; Frank et al., 2000; Martelli et al., 2017; Patton et al., 2000) in most of studies with an important look into clinical aspects, which are mostly based on the characteristics of the COP signal in response to different tasks. The prevailing spatial and temporal definitions of the margin of stability is simply an external manifestation of the genuine underlying control safety margin that the CNS adopts (e.g., the *GM* and *PM*, which are the characteristics of supraspinal control commands). Future studies are needed to disclose the relationship between intrinsic features of the safety margin (e.g., *GM*, *PM* which is studied here) and existing stability measures that were used in previous studies. In this regard, future works should examine the previously studied clinical/experimental tests through a computational biomechanical/neuromechanical model, evaluating *GM*/*PM* and other control engineering stability criteria with respect to the existing terms for the assessment of postural stability. Future studies can encompass complex models (e.g., double-inverted pendulum), as well as applying more general stability theorems (e.g., Lyapunov stability criterion (Khalil, 2015) for non-linear systems). Furthermore, this study was limited to quiet stance posturography. Basically, applying such fundamental concepts of theoretical stability in dynamic tasks (e.g., in the perturbed quiet stance and perturbed gait) provides a comprehensible explanation of patients’ balance performance. It should be emphasized that in dynamic as well as more challenging tasks such as the one-legged stance or standing on a narrow bar, other mechanisms of postural stability (e.g., hip strategy, and counter-rotating arm/trunk movement momentum) have their unique and considerable contributions (Hof, 2007; Tisserand et al., 2023), which should be taken into account in both model development and experimental data recordings. In these challenging tasks, COP posturography might not suffice alone, and other motion capture techniques might be required as well. In addition, the neuromechanical model used in this study, exclusively captures the biomechanical/physical and neuromechanical attributes of the human postural control which are reflected through the control mechanism and the control commands issued by the CNS, but not the aspects of sensory organization or the percent of sensory re-weighting and sensory integration due to different sensory conditions, as well as the cognitive influence of PD on patients’ postural control. Future studies are needed to disclose the unique contributions of each postural control modality, such as cognitive processes, sensory re-weighting algorithms, feedback time delay, CNS processing delay, and the different reactive or anticipatory movement strategies in voluntary tasks, through more complex models such as the well-established independent-channel model for sensory integration proposed by Peterka (Peterka, 2002) or the models reviewed by Chiba et al. (Chiba et al., 2016) or Olsson et al. (Olsson et al., 2021). Finally, yet importantly, future studies carrying longer training programs with follow-up inspection, accompanied by more time points of assessment, or enjoying diversity in training regimens are highly recommended. Future studies can investigate the problem of postural instability in other patient groups with instability (e.g., multiple sclerosis) or even aging people, using the stability measures proposed here.

## 5 Conclusion

This was the first study, to our best knowledge, which clinically utilized the Nyquist criterion (the concepts of gain margin (|*GM*|) and phase margin (*PM*) from control theory) to quantify the stability in a group of people with PD. The results showed the discriminating power of |*GM*| and *PM* in studying postural instability in PD, with clinical implications and applicability. Patients had a smaller margin of stability (i.e., |*GM*| and *PM*) compared with HCs. Particularly, a smaller *PM* in patients emphasized the abnormal delayed-response in PD. Balance training improved the stability margin in PD. |*GM*| improved early during the first weeks of training followed by a plateau. In contrast, *PM* showed late and continuous improvement. These observed trends of improvement provided recommendations for a strength training focus in the late sessions, as well as a continuous focus on timing exercises throughout the whole training program. |*GM*| and *PM* improved mainly on account of developed strength and reduction in time delay, respectively. Taken together, this study showed that examining fundamental stability theorems/criteria such as the Nyquist criterion (*GM* and *PM*), which inherently considers the dynamics of the human system and the CNS, in the analysis of balance performance in different clinical and experimental tasks, is a promising technique, which paves the path for the standardized quantification of stability on a unified and coherent ground. Such studies will link current clinical and experimental balance tests to each other and to their basic underlying governing rules.

## Data Availability

The raw data supporting the conclusion of this article will be made available by the authors, without undue reservation.

## Abbreviations

PD, Parkinson’s disease; HCs, healthy control subjects; COP, center-of-pressure; COM, center-of-mass; BOS, base of support; GM, gain margin; PM, phase margin; EO, eyes open; EC, eyes closed; RO, rigid surface with eyes open task; RC, rigid surface with eyes closed task; FO, foam surface with eyes open task; FC, foam surface with eyes closed task; F-tasks, foam-surface tasks; TUG, Timed Up and Go test; FRT, Functional Reach Test; BBS, Berg balance scale; PRE, progressive resistance exercise; LOS, Limit of Stability; FRF, frequency response function; CNS, central nervous system; PID controller, Proportional-integral-derivative controller.
