# Supplementary material for: Margins of postural stability in Parkinson’s disease: an application of control theory
Source: Front Bioeng Biotechnol. 2023 Sep 14;11:1226876. doi: 10.3389/fbioe.2023.1226876 (PMC10539597; doi:10.3389/fbioe.2023.1226876)
Supplement: Supplementary file 1 [file DataSheet1.docx]

Supplementary Material

Margins of Postural Stability in Parkinson’s Disease: an Application of Control Theory

Zahra Rahmati^1^, Saeed Behzadipour^1,2*^, Ghorban Taghizadeh^3^

^1^ *Mechanical Engineering Department, Sharif University of Technology, Tehran, Iran*

^2^ *Djawad Movafaghian Research Center in Neurorehab Technologies, Sharif University of Technology, Tehran, Iran*

^3^ *Rehabilitation Research Center, Department of Occupational Therapy, School of Rehabilitation Sciences, Iran University of Medical Sciences, Tehran, Iran*

*** Correspondence:**Corresponding Author
[behzadipour@sharif.edu](mailto:behzadipour@sharif.edu)

# Supplementary Appendix

The transfer function for the linearized inverted-pendulum system around the zero-degree state (i.e., assuming $\sin\left( \theta\right)\cong\theta$ for small values of $\theta$ around zero) in the frequency-domain (also known as the Laplace transform of a system equation) is as Eq.1. The transfer function for the proportional-integral-derivative controller (PID controller) comes in Eq.2. Finally, Eq.3 gives the transfer function of the open-loop system ($L(s)$) (i.e., without considering the unity feedback), which is the multiplication of the transfer function for the pendulum system and the one for the controller, in a linear system scheme.

$Pendulum (s) = \frac{1}{(Js^{2}-m_{B}gh)}$ (A1)

$PID \left( s \right)=\left( K_{P}+K_{D}\frac{s}{\left( 1+\frac{1}{N}s \right)} + \frac{K_{I}}{s} \right)e^{-\tau_{d}s}$ (A2)

$L(s) = Pendulum(s) * PID(s)$ (A3)

where *s* is the Laplace transform variable, *J*, *m*_B_, and *h* are the subject-specific system parameters (as described in Fig.1), and *g* = 9.81; *K_P_*, *K_D_*, and *K_I_*, are the control parameters, *τ*_d_ is the time delay, and *N* is usually a very large number (here taken as *N* = 575.5), which plays as a low-pass filter to makes the derivative term of the PID controller casual in addition to limiting high-frequency noise. Considering $L(s)$ in all ranges of frequency, that is, considering $L(j\omega)$ (taking the Laplace variable as $s = j\omega$, with frequencies $\omega=\left( -\infty, + \infty\right)$) brings us the frequency-response function of the open-loop system (also called a Bode diagram) from which the *GM* and *PM* are calculated in Eq.4-7.

*GM calculation*:

*GM* (Eq. 4) is defined as the negative logarithmic magnitude of system frequency-response (i.e., $L(j\omega)$) at the ‘phase crossover frequency ($\omega_{pc}$) (Eq. 5)’ (Ogata, 2010):

$phase crossover frequency \left( \omega_{pc} \right) \triangleq where \angle L\left( j\omega_{pc} \right)= -180^{\circ}$ (A4)

$GM = -\left| L\left( j\omega_{pc} \right) \right|$ (in decibel (dB)) (A5)

where $j=\sqrt{-1}$ is the unit imaginary number, operator $\angle$ gives the angular value of the complex number $L\left( j\omega_{pc} \right)$, ($L\left( j\omega_{pc} \right)$ is the frequency-response of the system at frequency $\omega_{pc}$), and the magnitude of $L\left( j\omega_{pc} \right)$ is in decibels (dB), i.e., 20*log_10_($|L\left( j\omega_{pc} \right)|$). The absolute value of *GM* (denoted by |*GM*|) is considered in this study.

*PM calculation*:

*PM* (Eq. 7) is defined as the difference between the angular value of system frequency-response ($\angle L(j\omega)$) at the ‘gain crossover frequency ($\omega_{gc}$) (Eq. 6)’, and the value -180⁰ (Ogata, 2010):

$gain crossover frequency \left( \omega_{gc} \right) \triangleq where \left| L\left( j\omega_{gc} \right) \right|= 0 dB$ (A6)

$PM = \angle L\left( j\omega_{gc} \right) + 180$ (in degree) (A7)
